# Divergent group-specific pathways of microbial diversity and network complexity maintain soil multifunctionality during alpine grassland restoration

**DOI:** 10.3389/fpls.2026.1923179

**Published:** 2026-08-19

**Authors:** Chenfeng Zheng, Mo Chen, Keyi Li, Yang Hu, Jiajia Wang, Kaihui Li, Jianqin Zhou, Xinping Zhu, Hongtao Jia

**Affiliations:** 1 College of Resources and Environment, Xinjiang Agricultural University, Urumqi, China; 2 Xinjiang Key Laboratory of Soil and Plant Ecological Processes, Xinjiang Agricultural University, Urumqi, China; 3 College of Grassland Science, Xinjiang Agricultural University, Urumqi, China; 4 Xinjiang Disaster Warning & Ecological Restoration Center, Xinjiang Agricultural University, Urumqi, China; 5 Bayinbuluk Grassland Ecosystem Research Station, Xinjiang Institute of Ecology and Geography, Chinese Academy of Sciences, Bayinbuluk, China; 6 College of Bioscience and Resources Environment, Beijing University of Agriculture, Beijing, China; 7 Key Laboratory of Urban Agriculture (North China), Ministry of Agriculture and Rural Affairs, Beijing, China; 8 College of Life Sciences, Xinjiang Agricultural University, Urumqi, China

**Keywords:** alpine grassland, microbial community assembly, microbial network complexity, natural restoration, protists, soil multifunctionality

## Abstract

**Introduction:**

Soil microorganisms maintain ecosystem multifunctionality, yet the group-specific pathways linking microbial diversity, community assembly, and network complexity to soil multifunctionality (SMF) during natural restoration of abandoned alpine grasslands remain unclear.

**Methods:**

We examined artificial grassland (AG) and 3-, 12-, and 31-year abandoned grasslands (AB03, AB12, and AB31) in Bayinbuluk, Xinjiang, and analyzed bacterial, fungal, and protistan diversity, community assembly, and co-occurrence networks in relation to SMF.

**Results:**

SMF was significantly higher at AB12 and AB31 than at AG. Stochastic processes dominated community assembly in all three groups: homogenizing dispersal in bacteria, drift in fungi, and in protists a shift from dispersal limitation at AB03 (90%) to drift at AB12 (100%). Bacterial network complexity was lowest at AB03, fungal complexity increased at AB31, and protistan complexity was unchanged. Partial least squares path modeling (PLS-PM) revealed divergent microbial pathways: fungal diversity and bacterial and protistan network complexity were positively associated with SMF, whereas fungal network complexity and protistan diversity showed negative associations.

**Discussion:**

Microbial–SMF linkages thus reflect a strategy of diversity dependence in fungi versus network dependence in bacteria and protists, indicating that assessing alpine grassland restoration requires group-specific rather than universal microbial indicators.

## 1 Introduction

Alpine grasslands are located in climatic transition zones, where degradation and restoration processes are constrained by both natural environmental variation and human activities (e.g., reclamation, grazing, and artificial seeding) (Harris, 2010; Wu et al., 2022). The Bayinbuluk alpine grassland in Xinjiang, the largest in China, is distinguished by severe cold and chronic nutrient scarcity: with a mean annual temperature of −4.8 °C and snow cover for nearly half of the year, the growing season is restricted to barely four months and soil nutrient mineralization is suppressed for most of the year (Zheng et al., 2026). In such systems, natural restoration after abandonment is a widely used, low-intervention, and cost-effective approach for degraded alpine grasslands (Seabloom et al., 2021). As restoration duration extends, soil physicochemical properties gradually evolve, providing an ideal time-series platform for investigating the reconstruction mechanisms of belowground ecosystem functioning (Zhang et al., 2024). However, single-function indicators cannot fully capture ecosystems restoration status across multiple biogeochemical dimensions. Ecosystem multifunctionality offers a holistic alternative by integrating carbon, nitrogen, phosphorus cycling, microbial biochemical processes, and nutrient supply into a unified framework (Garland et al., 2021; Luo et al., 2023). Therefore, using abandonment duration as an external environmental driver and ecosystem multifunctionality as a comprehensive response dimension provides a process-level framework for evaluating ecosystem sensitivity and its regulatory mechanisms under environmental change. Nevertheless, existing studies have largely focused on single restoration stages or short-term observations, neglecting the dynamic integration of functional changes across long-term successional sequences (Zheng et al., 2023; Meng et al., 2026). This temporal fragmentation limits a systematic understanding of how soil environment change drives the reorganization of multifunctionality over long restoration sequences.

Soil microorganisms are central to terrestrial ecosystem functioning, participating directly or indirectly in organic matter decomposition, nutrient mineralization, and soil organic carbon turnover. They are frequently invoked to explain the spatiotemporal variation in soil multifunctionality (SMF) (Falkowski et al., 2008; Soliveres et al., 2016; Bastida et al., 2021). Although numerous studies have examined the relationship between microbial diversity and SMF, results vary with environmental conditions and spatial scales (Wagg et al., 2019; Garland et al., 2021). During the restoration of alpine ecosystems, changes in microbial diversity may lag behind vegetation and soil nutrient recovery, and SMF enhancement does not always depend on increased diversity (Zhai et al., 2024). Species richness and taxonomic composition alone thus cannot fully explain how microorganisms regulate SMF recovery; analyses must shift from static descriptions of species abundance to community assembly mechanisms and interaction structures (Chen et al., 2022). In addition to bacteria and fungi, protists serve as important regulators of soil microbial food webs, influencing bacteria, fungi, and their associated functional processes through predation, resource competition, and trophic cascade effects (Hu et al., 2025a). Because protists are generally sensitive to moisture, pore connectivity, and prey resource availability, their community assembly and network structure may exhibit response patterns distinct from those of bacteria and fungi (Luan et al., 2020). Previous studies have shown that compositional changes and co-occurrence network characteristics of bacteria, fungi, and protists may jointly influence SMF during restoration (Kang et al., 2025), yet systematic comparisons of the relative pathways through which the three microbial groups act during natural restoration after abandonment remain lacking.

Within the null model framework, deterministic processes and stochastic processes jointly govern microbial community composition, diversity, and interaction networks (Stegen et al., 2012; Zhou and Ning, 2017). During abandonment restoration, changes in soil nutrients, pH, moisture conditions, and vegetation inputs may alter the relative importance of different assembly processes, thereby influencing community phylogenetic structure, functional redundancy, and niche differentiation (Louca et al., 2018; Zhou et al., 2025). However, restoration does not necessarily manifest as a unidirectional shift from stochastic to deterministic processes. Different microbial groups may exhibit distinct changes in assembly mechanisms across restoration stages due to variations in body size, dispersal ability, life-history strategies, and resource utilization patterns. Therefore, the critical question is whether abandonment restoration alters the relative contributions of assembly processes for bacterial, fungal, and protistan communities, and whether these changes are associated with the differentiated responses of network structure and SMF.

Microbial co-occurrence networks describe potential interaction patterns from the perspective of community association structures, providing complementary information for understanding functional regulatory mechanisms beyond microbial diversity (Wagg et al., 2019; Wang et al., 2024). Network complexity, particularly node connectivity and modularity, has proven more effective than single diversity indices in predicting nutrient cycling, enzyme activity, and multifunctionality maintenance (Zhai et al., 2024; Xiao et al., 2025). In grassland restoration studies, increased network complexity significantly enhances SMF, whereas network degradation weakens functional redundancy and complementarity (Wagg et al., 2019; Xiao et al., 2025). However, current grassland restoration research has focused primarily on bacteria and fungi, with protists rarely incorporated into the same network analysis framework (Thakur and Geisen, 2019). Predatory protist groups can alter microbial food web structure by regulating bacterial and fungal communities, while saprotrophic or photosynthetic groups may also participate in soil processes through resource utilization and nutrient supplementation. Although the relationships among microbial diversity, community assembly, co-occurrence networks, and SMF have received increasing attention, whether the three microbial groups relate to SMF through convergent or divergent pathways under the natural restoration gradient of abandoned alpine grasslands remains unclear. Evaluating restoration outcomes is now a routine task in grassland management, yet conventional vegetation- and soil-based metrics respond slowly to belowground recovery. Microbial attributes are promising indicator candidates; however, if bacteria, fungi, and protists recover asynchronously, a single generalized microbial metric may not adequately represent overall recovery, and monitoring frameworks would therefore benefit from taxon-specific indicators.

Accordingly, we selected artificial grassland (AG) and 3-, 12-, and 31-year abandoned grasslands (AB03, AB12, and AB31) in the Bayinbuluk alpine grassland, Xinjiang, to systematically analyze the diversity, assembly processes, and co-occurrence network structure of bacterial, fungal, and protistan communities and their relationships with SMF. The specific objectives were as follows: (i) to compare the changes in diversity, community composition, assembly processes, and network structure among bacteria, fungi, and protists across different restoration stages; (ii) to evaluate whether the associations between diversity and network attributes of different microbial groups and SMF are group-specific; and (iii) to examine whether community assembly is indirectly linked to SMF through microbial diversity and network structure, and whether these inferred pathways differ among microbial groups. Based on existing theories and preliminary evidence, we propose the following hypotheses: (H1) the explanatory power of microbial network complexity relative to single diversity indices for SMF is group-specific, with diversity outweighing network complexity for fungi, and network complexity outweighing diversity for bacteria and protists; and (H2) shifts in community assembly processes during natural restoration are indirectly linked to SMF through their effects on microbial diversity and network structure, with these cascading pathways differing among microbial groups.

## 2 Materials and methods

### 2.1 Overview of the study area

The study area was located in the Bayinbuluk alpine grassland, Xinjiang, the largest alpine grassland in China. The experimental sites were established in Baxilige Village, Bayinbuluk Town (42°48.66′–42°49.08′ N, 84°29.10′–84°30.54′ E; **Supplementary Figure 1**), at an elevation of 2300–3042 m. This region has a typical alpine climate, with a mean annual temperature of −4.8 °C, annual precipitation of 273 mm, and a snow cover period of 150–180 days. The soil type is subalpine steppe soil, with loess as the parent material. The dominant plant species are *Carex melanantha* and *Elymus dahuricus*, and the growing season typically extends from mid-May to early September (Zheng et al., 2026). This study employed a space-for-time substitution approach, selecting artificial grassland (AG) and 3-, 12-, and 31-year abandoned grasslands (AB03, AB12, and AB31) within the same region as a restoration stage gradient. To minimize confounding between site background and abandonment duration (Walker et al., 2010), all sites share the same climatic conditions, soil type, and parent material, and the three abandoned grasslands had comparable land-use histories: all were originally natural grasslands that had been grazed or reclaimed and subsequently abandoned, with natural restoration beginning in 2022 (AB03), 2013 (AB12), and 1994 (AB31). The artificial grassland was a cultivated grassland established in recent years (Li et al., 2023).

### 2.2 Soil sample collection and analysis

Mid-August coincides with the seasonal peak of aboveground biomass and soil microbial activity in this system, and temperature and precipitation during the sampling period were within the normal range of the local climate record (1984–2024; **Supplementary Figure 2**). In mid-August 2025, five replicate quadrats (2 m × 2 m) were randomly established in each of AG, AB03, AB12, and AB31, with inter-quadrat distances greater than 5 m and distances from fences exceeding 5 m to minimize edge effects, totaling 20 quadrats. Within each quadrat, topsoil (0–20 cm) was collected using a five-point composite sampling method and thoroughly mixed after removal of visible plant roots, stones, and litter. The composite soil samples were divided into three portions: one was flash-frozen in liquid nitrogen and stored at −80 °C for microbial molecular analysis; one was air-dried for soil physicochemical property determination; and the remaining portion was stored at 4 °C for soil enzyme activity and inorganic nitrogen content determination (Zheng et al., 2026).

Soil pH was measured using a pH meter (BPH-7100, BELL Analytical Instruments, Dalian, China). Soil organic carbon (SOC) was determined by potassium dichromate oxidation. Total nitrogen (TN) was analyzed using an elemental analyzer (VARIO EL III, Hanau, Germany). Total phosphorus (TP) and available phosphorus (AP) were measured using a UV spectrophotometer (UV-1800, Mapada, Shanghai, China), with AP extracted by sodium bicarbonate and determined by the molybdenum-antimony colorimetric method. Total potassium (TK) was determined by sodium hydroxide fusion–flame photometry. Available nitrogen (AN) was determined by the alkaline diffusion method. Available potassium (AK) was extracted with ammonium acetate and measured by flame photometry. Ammonium nitrogen (NH_4_^+^-N) and nitrate nitrogen (NO_3_⁻-N) were determined using a continuous flow analyzer (AA3, Bran+Luebbe, Germany). The activities of β-1,4-glucosidase (BG), cellobiohydrolase (CBH), β-1,4-N-acetylglucosaminidase (NAG), and alkaline phosphatase (ALP) were measured using a fluorimetric microplate assay with 4-methylumbelliferyl (4-MUB)-labeled substrates. Urease (URE) activity was determined by the indophenol blue colorimetric method using urea as the substrate, measuring the amount of ammonium nitrogen released within 24 h. All enzyme activities were expressed as nanomoles of product per gram of dry soil per hour (nmol·g⁻¹·h⁻¹) (Li et al., 2024).

### 2.3 Quantification of soil multifunctionality

Soil multifunctionality was assessed based on 14 functional indicators, including soil organic carbon (SOC), total nitrogen (TN), total phosphorus (TP), total potassium (TK), inorganic nitrogen (NH_4_^+^-N and NO_3_⁻-N), available nitrogen (AN), available phosphorus (AP), available potassium (AK), and extracellular enzyme activities: β-glucosidase (BG), cellobiohydrolase (CBH), urease (URE), β-N-acetylglucosaminidase (NAG), and alkaline phosphatase (ALP). All variables were standardized to the 0–1 range prior to analysis to eliminate dimensional differences. Three complementary approaches were employed to quantify multifunctionality: (i) Mean-based multifunctionality index (MFI): the arithmetic mean of standardized indicators, reflecting overall functional levels. (ii) Multidimensional multifunctionality (MDF): principal coordinate analysis (PCoA) based on Euclidean distances of standardized data, with the first three axes (Dim1–Dim3) used to characterize the main gradients of soil multifunctionality; the direction of each axis was determined by the functional indicators with the highest loadings (**Supplementary Figure 3**). (iii) Multi-threshold multifunctionality: following the multi-threshold approach (Byrnes et al., 2014), thresholds of 10%, 25%, 50%, 75%, and 90% of the maximum value of each indicator were used to calculate the proportion of functional indicators exceeding each threshold. These five thresholds span the gradient from lenient to stringent functional performance, avoiding the arbitrariness of single-threshold assessment and allowing threshold-dependent effects to be detected. The 10% and 25% thresholds represent low functional levels, the 50% and 75% thresholds medium levels, and the 90% threshold a high level. The area under the threshold–function proportion curve (MUF) was calculated as a comprehensive metric integrating performance across all thresholds (Chen et al., 2026).

### 2.4 DNA extraction, amplification, sequencing and data processing

High-throughput amplicon sequencing was employed to characterize the diversity and composition of soil bacterial, fungal, and protistan communities (Xu et al., 2021). The V4–V5 region of the bacterial 16S rRNA gene was amplified using primers 515F/907R; the fungal ITS1 region was amplified using primers ITS1F/ITS2; and the V4 region of the protistan 18S rRNA gene was amplified using primers TAReuk454FWD1 (5′-CCAGCASCYGCGGTAATTCC-3′)/TAReukREV3 (5′-ACTTTCGTTCTTGATYRA-3′). Each sample was subjected to three independent PCR reactions in a 25 μL reaction system: 12.5 μL 2× Phusion Master Mix, 1.0 μL each of forward and reverse primers (10 μM), and 10 ng template DNA. PCR conditions were as follows: 98 °C for 30 s; 25–30 cycles (98 °C for 10 s, 55 °C for 30 s, 72 °C for 30 s); and final extension at 72 °C for 5 min. Replicate amplicons were pooled, purified by agarose gel electrophoresis, precisely quantified using Qubit, and combined in equimolar amounts for library construction. Paired-end sequencing (2 × 250 bp) was performed on the Illumina NovaSeq 6000 platform (Xu et al., 2021).

Raw data were processed in QIIME 2 using DADA2, including quality filtering (Q ≥ 20), paired-end merging, chimera removal, and denoising to obtain amplicon sequence variants (ASVs). After removal of mitochondrial, chloroplast, and non-target sequences, bacterial, fungal, and protistan ASVs were annotated against the SILVA 138.1, UNITE v9.0, and PR2 v5.0.0 databases (Guillou et al., 2013), respectively; for functional interpretation, protistan ASVs were further assigned to three broad trophic modes at the phylum level (Adl et al., 2019; Oliverio et al., 2020): predatory (Ciliophora), phototrophic (Ochrophyta), and parasitic (Oomycota and Apicomplexa). A distinct saprotrophic mode could not be resolved, as no detected phylum is predominantly saprotrophic and V4 resolution is insufficient below the phylum level. All raw sequencing data have been deposited in the NCBI Sequence Read Archive under the BioProject accession number PRJNA1501858.

### 2.5 Construction of microbial co-occurrence networks

Co-occurrence networks for bacteria, fungi, and protists were constructed separately using R (v. 4.5.0). To minimize the influence of rare ASVs and low-abundance noise on network construction, ASVs with mean relative abundance below 0.05% were first removed. Spearman rank correlation analysis was then employed to calculate pairwise correlations among ASVs, and significant associations were defined as those with |ρ| > 0.7 and FDR-corrected *P* < 0.05 using the Benjamini-Hochberg method, yielding overall co-occurrence networks for bacteria, fungi, and protists, respectively. To obtain sample-level network topological characteristics, subnetworks were extracted from the overall network for each sample. Specifically, if an ASV had a relative abundance greater than 0 in a given sample and belonged to the nodes of the overall network, it was retained as a node of the sample subnetwork; if both end nodes of a significant association edge in the overall network were present in that sample, the edge was retained. Isolated nodes were subsequently removed, and topological indices of the sample subnetworks were calculated. Seven indices were computed for each sample subnetwork: number of nodes, number of edges, average degree, clustering coefficient, average path length, graph diameter, and graph density. The network complexity index was then calculated using these topological features via multidimensional scaling analysis; before this calculation, the average path length and diameter were transformed to their reciprocals (x^-1^) (Manning et al., 2018; Xiao et al., 2025). This composite index was used to represent the overall topological complexity of each subnetwork, while individual indices were retained to interpret the structural components underlying the composite pattern (Barberán et al., 2012; Xiao et al., 2025).

### 2.6 Quantification of microbial community assembly processes

Microbial community assembly processes were quantified using the β-nearest taxon index (βNTI) and the Raup–Crick index (RC_bray_) (Xun et al., 2019). For each microbial group, representative ASV sequences were aligned using MAFFT, and phylogenetic trees were inferred using FastTree with midpoint rooting via the q2-phylogeny plugin in QIIME 2 (Price et al., 2010; Standley and Katoh, 2013). FastTree applies a GTR+CAT substitution model (Price et al., 2010). For fungi, whose ITS-based phylogenies are poorly resolved, stochasticity was additionally assessed using the tree-free taxonomic normalized stochasticity ratio (Ning et al., 2019). Based on the ASV tables and the rooted phylogenetic trees, βNTI was calculated using the iCAMP package (Ning et al., 2020): abundance-weighted β-mean nearest taxon distance (βMNTD) was computed, null communities were generated with the independent-swap algorithm (999 randomizations), and βNTI was obtained as the standardized difference between the observed and null βMNTD distributions. RC_bray_ was calculated based on Bray-Curtis dissimilarities using the same null-model procedure. |βNTI| > 2 indicated deterministic processes, with βNTI > 2 representing heterogeneous selection and βNTI < −2 representing homogeneous selection; |βNTI| < 2 indicated stochastic processes. Stochastic processes were further partitioned using RC_bray_: dispersal limitation was assigned when |βNTI| < 2 and RC_bray_ > 0.95; homogenizing dispersal when |βNTI| < 2 and RC_bray_ < −0.95; and drift when |βNTI| < 2 and |RC_bray_| ≤ 0.95. The relative contributions of assembly processes across restoration stages were subsequently calculated (Jiao et al., 2022).

### 2.7 Statistical analysis

All statistical analyses and visualizations were performed in R (v. 4.5.0). One-way analysis of variance (ANOVA) was used to examine differences in soil functional indicators, microbial α-diversity, network topological indices, and SMF among restoration stages; when significant differences were detected, Tukey’s HSD *post-hoc* test was employed for multiple comparisons. Microbial α-diversity was assessed using the richness and Shannon indices. For protists, richness, Shannon diversity, and relative abundance were additionally calculated for each trophic mode, and their relationships with SMF indices were examined using Spearman correlations. Community β-diversity for bacteria, fungi, and protists was calculated based on Bray-Curtis distances and visualized through principal coordinate analysis (PCoA); differences in community composition were tested using PERMANOVA. Relative abundances of bacteria, fungi, and protists were summarized at the phylum level, and taxa with mean relative abundance below 1% were classified as “Others”. To evaluate direct and indirect relationships among abandonment duration, soil properties, microbial attributes, community assembly processes, and soil multifunctionality, partial least squares path modeling (PLS-PM) was performed for bacteria, fungi, and protists using the plspm package. Multicollinearity among the predictors of each inner-model equation was assessed using variance inflation factors, and residuals of the multifunctionality equations were checked for normality, heteroscedasticity, and independence (**Supplementary Table 1**).

## 3 Results

### 3.1 Changes in microbial diversity and community composition during the natural restoration of abandoned alpine grassland

**Figure 1:**
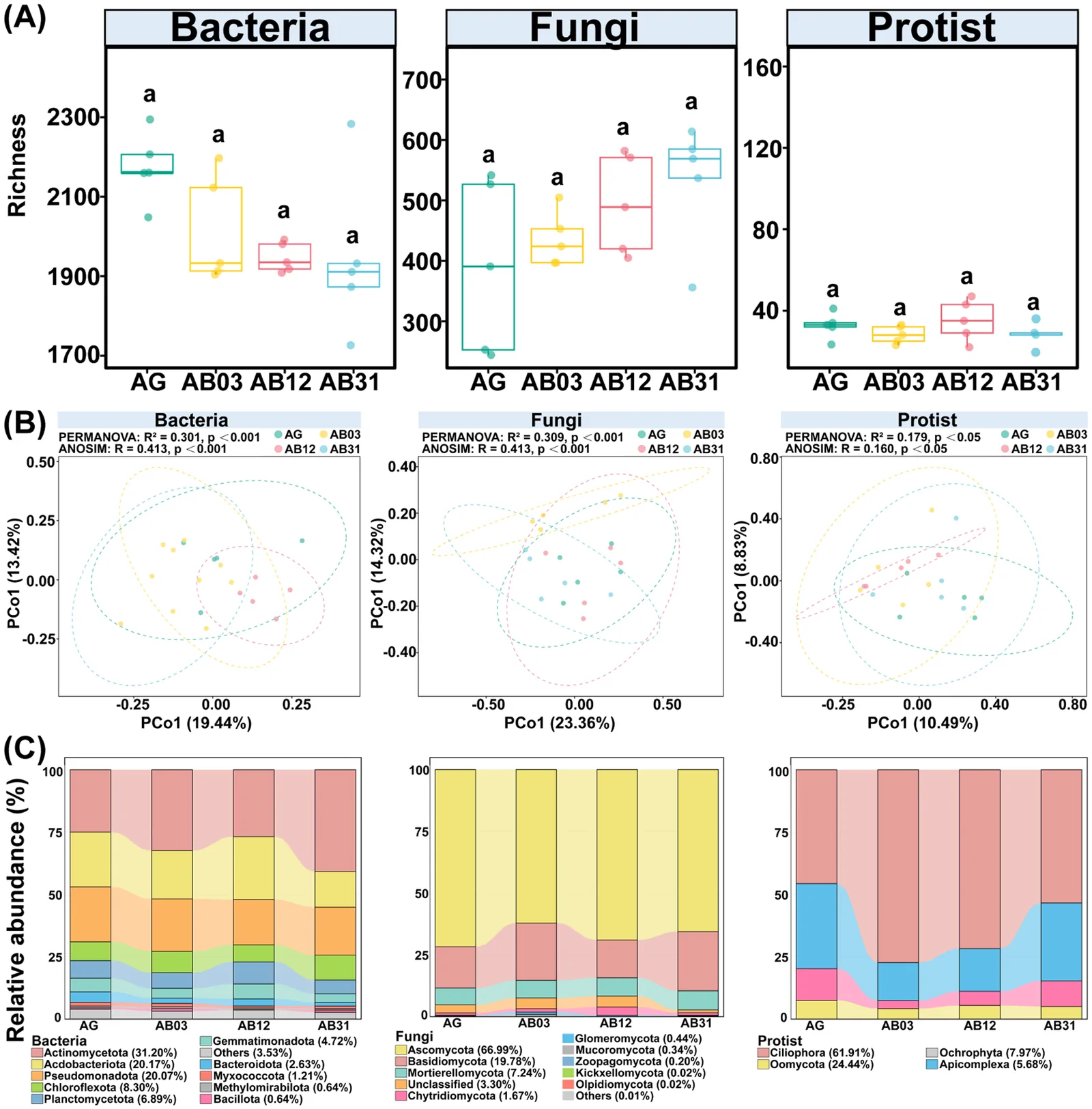
Changes in microbial diversity and community composition during natural restoration of abandoned alpine grassland. **(A)** Richness of bacteria, fungi, and protists across restoration stages. Different lowercase letters indicate significant differences among restoration stages within each microbial group (*P*<0.05). **(B)** Principal coordinate analysis (PCoA) based on Bray-Curtis dissimilarity, illustrating community compositional turnover (PERMANOVA, 999 permutations). **(C)** Relative abundance of dominant phyla for bacteria, fungi, and protists. Taxa contributing < 1% of average relative abundance were classified as “Others”.

During natural restoration, the diversity of the three microbial communities exhibited divergent responses. Regarding richness, bacterial richness showed a decreasing trend with increasing abandonment duration, fungal richness showed an increasing trend, and protistan richness remained relatively stable; however, no significant differences were observed among restoration stages (**Figure 1A**; **Supplementary Table 2**). For the Shannon index, neither bacteria nor fungi showed significant changes across restoration stages, whereas the protistan Shannon index was significantly higher in AG than in AB03 and AB31 (**Supplementary Figure 4**; **Supplementary Table 2**). In contrast to α-diversity, community composition of the three microbial groups displayed marked stage-specific differentiation. PCoA based on Bray–Curtis distances and PERMANOVA revealed that bacterial and fungal community compositions differed significantly along the restoration sequence (*P* < 0.001), while protistan community composition showed relatively weaker but still significant differentiation (*P* < 0.05; **Figure 1B**). At the phylum level, the relative abundance of Pseudomonadota in the bacterial community decreased with increasing abandonment duration (highest in AG, lowest in AB31), whereas Actinomycetota peaked in AB31; Acidobacteriota exhibited a hump-shaped pattern, reaching its maximum in AB12. In the fungal community, Basidiomycota relative abundance was lower in AB12 than in other stages. In the protistan community, Ciliophora was highest in AB03, while Oomycota was relatively more abundant in AG and AB31 (**Figure 1C**). At the trophic-mode level, the relative abundance of predatory protists (Ciliophora) increased from 45.6% in AG to 77.2% in AB03, whereas parasitic protists (Oomycota and Apicomplexa) were proportionally most abundant in AG (41.7%) and least abundant in AB03 (**Supplementary Figure 5**; **Supplementary Table 2**). RDA further indicated that environmental factors explained varying proportions of community composition among the three microbial groups (fungi, 24.95%; bacteria, 11.99%; protists, 2.49%; **Supplementary Figure 6**). Notably, the opposing trends of Pseudomonadota and Actinomycetota mirrored the shift in soil nitrogen forms, from NO_3_⁻-N-dominated conditions in AG to NH_4_^+^-N-enriched conditions in AB31. Overall, natural restoration after abandonment exerted stronger effects on microbial community composition than on α-diversity.

### 3.2 Changes in the processes of microbial community assembly during the natural restoration of abandoned alpine grassland

**Figure 2:**
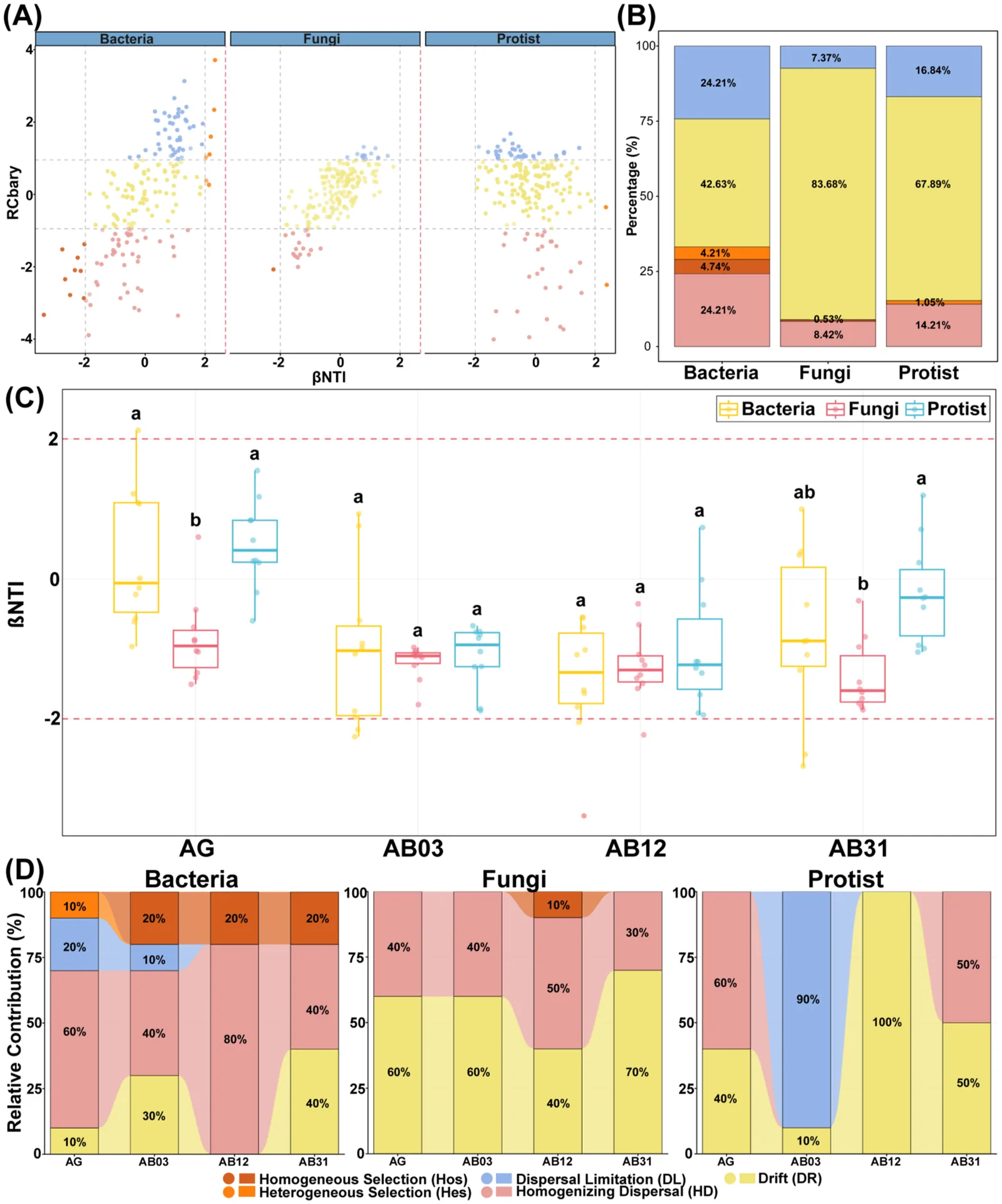
Variations in microbial community assembly processes during natural restoration of abandoned alpine grassland. **(A)** Relationship between β-nearest taxon index (βNTI) and modified Raup-Crick index (RC_bray_). **(B)** Relative contributions of deterministic and stochastic processes to the assembly of bacterial, fungal, and protistan communities. **(C)** Distribution of βNTI values for bacterial, fungal, and protistan communities across restoration stages. Dashed lines indicate the |βNTI| = 2 threshold. Different lowercase letters indicate significant differences among restoration stages within each microbial group (*P*<0.05). **(D)** Relative contributions of deterministic and stochastic processes to community assembly based on null model quantification.

Null-model analysis based on βNTI and RC_bray_ revealed that community assembly was dominated by stochastic processes in all three microbial groups, but the composition of their processes differed among groups (**Figures 2A, B**). Overall, drift contributed most to fungal assembly (83.68%) and least to bacterial assembly (42.63%); dispersal limitation contributed more to protistan than to fungal assembly, while bacteria showed equal contributions from dispersal limitation and homogenizing dispersal (both at 24.21%; **Figure 2B**).

At AG and AB31, |βNTI| was significantly lower for fungal communities than for bacterial and protistan communities (*P* < 0.05; **Figures 2A, C**), indicating more consistently stochastic assembly in fungal communities at these stages. The relative contributions of assembly processes exhibited pronounced group specificity across restoration stages. Homogenizing dispersal contributed substantially to bacterial community assembly, reaching 80% in AB12; meanwhile, dispersal limitation occurred mainly in AG and AB03 (**Figure 2D**). Fungal communities were dominated by drift across all restoration stages, with homogeneous selection appearing only in AB12 at 10%. Consistent with this, tree-free tNST also indicated stochasticity dominance at all stages (**Supplementary Table 3**). Protistan community assembly showed the most pronounced stage-dependent changes: AG and AB31 were jointly governed by homogenizing dispersal and drift, AB03 was characterized by 90% dispersal limitation, and AB12 was completely dominated by drift (100%) (**Figure 2D**). Thus, although stochastic processes dominated assembly in all three groups, their composition was group-specific and, for protists, strongly stage-dependent.

### 3.3 Changes in microbial co-occurrence networks and their complexity during the natural restoration of abandoned alpine grassland

**Figure 3:**
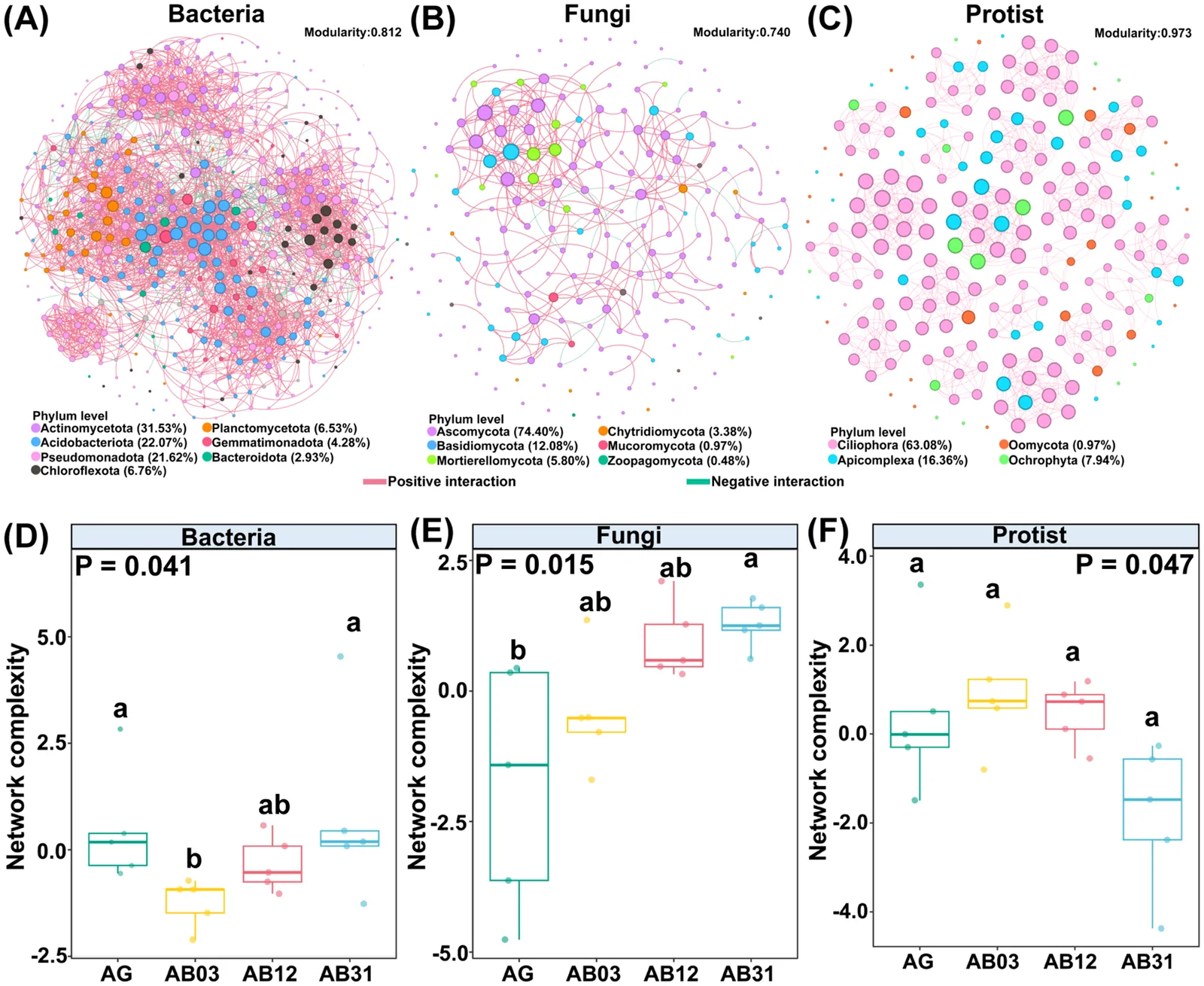
Dynamics of microbial co-occurrence network complexity during natural restoration of abandoned alpine grassland. **(A–C)** Co-occurrence networks of bacteria, fungi, and protists. Node colors represent major phyla; edge colors indicate positive (red) or negative (blue) Spearman correlations (|ρ| > 0.7, FDR-corrected *P* < 0.05). **(D–F)** Network complexity indices for bacterial, fungal, and protistan networks. Different lowercase letters above boxplots indicate significant differences among treatments (*P* < 0.05, Tukey’s HSD test).

Co-occurrence network structures differed markedly among microbial groups during natural restoration. Modularity was highest in the overall protistan network (0.973) and lowest in the fungal network (0.740). In terms of major node composition, the bacterial network was primarily composed of Actinomycetota, Acidobacteriota, and Pseudomonadota; the fungal network was dominated by Ascomycota and Basidiomycota; and the protistan network was dominated by Ciliophora and Apicomplexa (**Figures 3A–C**). None of the complexity indices for bacteria showed significant changes across restoration stages (**Supplementary Table 4**). In the fungal network, only average path length and diameter were significantly higher in AB12 and AB31 than in AG (**Supplementary Table 4**). In the protistan network, average degree and graph density were significantly higher in AB03 than in AB31, with no significant differences detected for the remaining indices (**Supplementary Table 4**). The composite network complexity index further revealed that bacterial network complexity was significantly higher in AG and AB31 than in AB03 (**Figure 3D**); fungal network complexity was significantly higher in AB31 than in AG (**Figure 3E**). Although overall ANOVA indicated a trend of stage-dependent differences in protistan network complexity, *post-hoc* comparisons did not reveal clear restoration-stage differentiation (**Figure 3F**). Collectively, natural restoration of abandoned alpine grassland exerted more pronounced effects on bacterial and fungal network complexity, whereas protistan networks remained relatively stable.

### 3.4 Changes in soil multifunctionality during the natural restoration of abandoned alpine grassland

Along the natural restoration gradient, 14 soil functional indicators exhibited divergent response patterns (**Supplementary Figure 8**). SOC and TN peaked in AB03, significantly higher than in AG; TP showed no significant differences, whereas TK was significantly lower in AB31 than in AG and AB03. Available nutrients (AN, AP, AK) were significantly higher in AB12 than in AG, but were at their lowest levels in AB03. NH_4_^+^-N was highest in AB31, whereas NO_3_⁻-N was highest in AG and lowest in AB03. BG, CBH, and ALP were all lowest in AG, with BG and CBH significantly lower than in the other three stages; NAG and URE showed no significant differences across restoration stages (**Supplementary Figure 8**).

**Figure 4:**
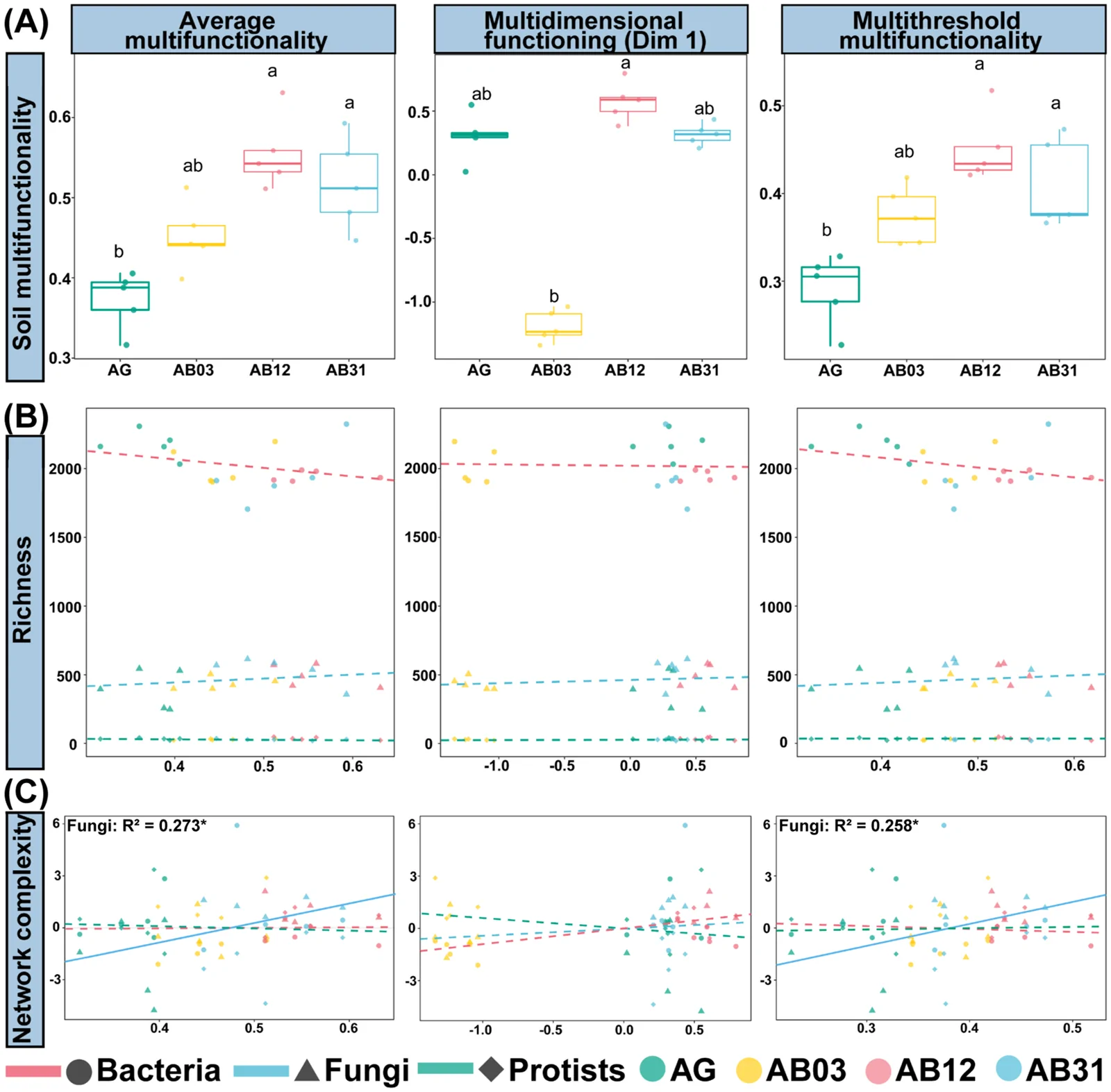
Soil multifunctionality across restoration stages and its relationships with microbial attributes. **(A)** Mean-based multifunctionality index (MFI), multidimensional multifunctionality (MDF, Dim1), and multi-threshold multifunctionality (MUF). Different lowercase letters indicate significant differences among treatments (*P* < 0.05, Tukey’s HSD test). **(B)** Relationships between microbial richness and multifunctionality indices. **(C)** Relationships between microbial network complexity and multifunctionality indices. Asterisks denote significant Spearman correlations (*P < 0.05).

Comprehensive multifunctionality assessment revealed that MFI and MUF increased with abandonment duration: both AB12 and AB31 were significantly higher than AG (*P* < 0.05; **Figure 4A**). MDF was lowest in AB03, significantly lower than in AB12 (**Figure 4A**). Throughout the restoration sequence, richness of all three microbial groups showed no significant correlation with any multifunctionality index, including at the protistan trophic-mode level (**Figure 4B**; **Supplementary Table 5**); only fungal network complexity was significantly positively correlated with MFI and MUF (**Figure 4C**).

Multidimensional functional analysis showed that Dim1 (42.1%), Dim2 (29.3%), and Dim3 (10.2%) all exhibited significant stage-dependent differentiation: Dim1 was lowest in AB03, Dim2 lowest in AG, and Dim3 highest in AB12 (**Supplementary Figure 9**). Multi-threshold analysis revealed threshold-dependence of SMF: at low (10%–25%) and medium (50%–75%) thresholds, abandoned grasslands were generally significantly higher than AG, but no significant differences were detected at the 90% threshold (**Supplementary Figures 10A–E**). The area under the MUF curve peaked in AB12, with both AB12 and AB31 significantly higher than AG (**Supplementary Figure 10F**). These stage patterns were robust to alternative SMF algorithms (**Supplementary Table 6**; **Supplementary Figures 11A, B**). These results indicate that, under the cold alpine climate, natural restoration enhanced SMF only at low-to-medium functional levels: individual soil functions peaked at different stages rather than reaching high levels simultaneously.

### 3.5 Microbial pathways and environmental drivers of soil multifunctionality

**Figure 5:**
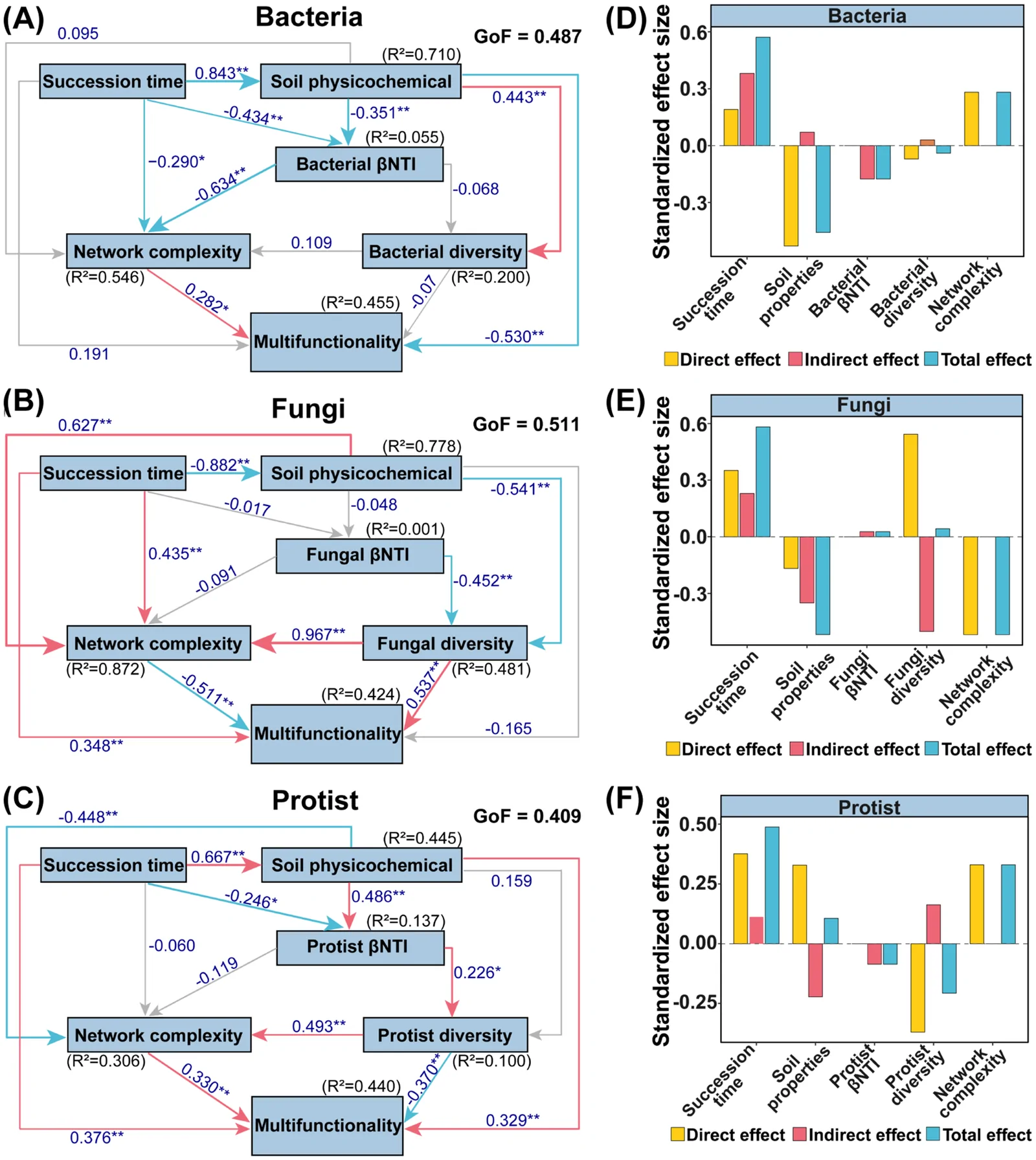
Multidimensional microbial attributes regulating soil multifunctionality as revealed by partial least squares path modeling (PLS-PM). **(A–C)** Path models for bacteria, fungi, and protists. Red arrows indicate positive significant paths, blue arrows indicate negative significant paths, and gray arrows indicate non-significant paths. Line width represents effect strength; R² values indicate the proportion of explained variance for endogenous variables. **(D–F)** Direct, indirect, and total effects of restoration duration, soil physicochemical properties, βNTI, diversity, and network complexity on soil multifunctionality. (*P < 0.05, **P < 0.01).

Partial least squares path modeling (PLS-PM) revealed that the pathways linking restoration duration, soil physicochemical properties, community assembly processes, microbial diversity, and network complexity to MFI exhibited pronounced group-specific differences, and these driver–SMF relationships were consistent across alternative SMF algorithms (**Supplementary Tables S6**, **S7**; **Supplementary Figure 11C**). All three models showed adequate fit, with GoF values of 0.511, 0.487, and 0.409 for the fungal, bacterial, and protistan models, respectively (**Figure 5**). In the bacterial model, restoration duration showed significant negative associations with soil physicochemical properties, βNTI, and network complexity, but its direct path to SMF was not significant. Soil physicochemical properties were significantly positively related to bacterial diversity, but significantly negatively related to βNTI and SMF; βNTI was further negatively associated with network complexity. Bacterial network complexity was significantly positively related to SMF, whereas the direct path from bacterial diversity to SMF was not significant (**Figure 5A**). In the fungal model, restoration duration was positively associated with network complexity and SMF, but negatively associated with soil physicochemical properties; diversity was positively related to both network complexity and SMF, whereas network complexity was negatively associated with SMF (**Figure 5B**). The two attributes were strongly correlated, and when their shared variance was apportioned between them, both contributed positively to SMF (**Supplementary Table 1**). In the protistan model, both restoration duration and soil physicochemical properties were significantly positively related to SMF; protistan diversity was negatively related to SMF, whereas network complexity was positively related to SMF (**Figure 5C**).

Effect decomposition further revealed that restoration duration exhibited positive total effects on SMF across all three models; the total effect directions of soil physicochemical properties and βNTI varied among microbial groups. Fungal diversity showed a weak positive total effect, whereas bacterial and protistan diversity showed negative or weak effects; network complexity exhibited positive total effects in the bacterial and protistan models, but a negative total effect in the fungal model (**Figures 5D–F**). Collectively, the association pathways between the three microbial groups and SMF showed clear differentiation: the fungal model primarily reflected a diversity-related pathway, whereas the bacterial and protistan models more strongly reflected network complexity-related pathways.

## 4 Discussion

### 4.1 Restoration responses and resource constraints of soil multifunctionality

Soil multifunctionality (SMF) did not increase linearly with restoration duration but rose significantly after 12 years of abandonment and remained elevated through 31 years. With a single site per stage, however, this pattern describes differences among stages rather than a measured trajectory, and may partly reflect pre-abandonment management differences (Walker et al., 2010). Moreover, the AG reference is a cultivated rather than abandoned grassland, so comparisons against it include a land-use contrast. Even so, the pattern indicates that increasing restoration duration does not necessarily correspond to synchronous enhancement across all soil functional dimensions. Ecosystem multifunctionality integrates multiple processes, and its dynamics depend on coordinated responses across multiple functional dimensions rather than on a single nutrient pool (Garland et al., 2021; Ma et al., 2026). Unlike findings from some grassland restoration studies where soil carbon accumulation and functional recovery are relatively synchronous (Saba et al., 2026), the long-term low-temperature alpine environment in our study area may slow organic matter decomposition and nutrient transformation, causing soil functional recovery to be constrained more by resource availability than by total supply (Yuan et al., 2025).

At AB03, SOC, TN, BG, and CBH were all significantly higher than in AG, but available nutrients (AN, AP, AK) were at their lowest levels, exhibiting a pattern of high organic matter pools but low available nutrient supply. This suggests that organic matter and some carbon-cycling enzyme activities had already accumulated in the early restoration stage, but their transformation into plant- and microbially available nutrient forms may remain insufficient (Zhu et al., 2021; Zhou et al., 2026). By AB12, available nutrients had markedly increased while total nutrients did not increase synchronously, further indicating that SMF improvement may depend more on amelioration of available nutrient supply capacity than on continuous accumulation of total nutrient indicators.

Multi-threshold analysis also supports this conclusion: natural restoration primarily enhanced the capacity of multiple functional indicators to reach low-to-medium levels, but was insufficient to drive most functional indicators to simultaneously reach high levels (Zheng et al., 2023). Moreover, MFI and MUF at AB31 were 7% and 9% lower than at AB12, respectively; although not significant, these declines suggest that restoration gains may be mainly concentrated in the early-to-middle stages. The preceding AG–AB12 gain, by contrast, was large, so the space-for-time uncertainty bears mainly on the late-stage shape rather than the mid-term gain. A mid-term peak followed by a significant decline has been reported on the Qinghai–Tibetan Plateau (Meng et al., 2026); with 19 unsampled years between AB12 and AB31, the present data cannot establish whether the slight decline here is the onset of a similar downturn or noise around a plateau. If genuine, this divergence may reflect the severe climate of the Tianshan alpine grassland, where low temperature and prolonged snow cover slow nutrient turnover and may prolong the recovery phase. That said, restoration duration remains a reasonable interpretation of the stage differences, which were consistent across independent SMF indices (**Figure 4A**; **Supplementary Figure 9**) and echo nonlinear recovery in other chronosequences. Verifying the inferred trajectory and its regional generality requires longer or denser time series—spanning multiple seasons and years—particularly long-term monitoring of permanent plots (Zheng et al., 2023; Meng et al., 2026). Overall, SMF recovery during abandonment depended more on the joint improvement of nutrient supply, extracellular enzyme activities, and multiple soil functions than on restoration duration or total nutrient accumulation. Management should therefore differ among stages: early-stage measures should promote the release of available nutrients from accumulated organic matter; mid-term sites should be protected from renewed disturbance to maintain peak multifunctionality; and late-stage sites should remain under monitoring, with measures to promote nutrient turnover considered only if the slight decline proves genuine.

### 4.2 Functional differentiation driven by microbial community assembly mechanisms

Natural restoration did not shift microbial assembly unidirectionally from stochastic to deterministic control; instead, it reallocated the relative importance of different stochastic processes in a group-specific manner. This differentiation is closely related to differences in dispersal ability, life-history strategies, and environmental sensitivity among the three microbial groups (Dini-Andreote et al., 2015; Xun et al., 2019). In bacterial communities, the contribution of homogenizing dispersal was high, reaching 80% at AB12, indicating that bacterial community composition became increasingly homogeneous among sites at this stage. Because AB12 concurrently exhibited high SMF and available nutrient levels, this pattern may reflect enhanced bacterial dispersal and exchange among sites as resources improved, which reduced spatial differences in community composition. Bacteria are small in size and have short generation times, making them more susceptible to passive dispersal via water flow, soil particle migration, and root activity, which provides an ecological explanation for their high contribution of homogenizing dispersal (Aguilar-Trigueros et al., 2022). However, community homogenization does not necessarily represent functional enhancement; excessively strong homogenization may also weaken potential functional complementarity among sites, and its effects on long-term functional stability require further verification (Yang et al., 2025). Indeed, within-group bacterial β-diversity was maintained across stages and uncorrelated with SMF (**Supplementary Figure 7**). Moreover, taxonomic homogenization does not necessarily imply functional homogenization, as dispersal among resource-improving sites may instead spread resource-cycling taxa (Mouquet and Loreau, 2003), which may partly explain the high SMF observed at AB12.

Unlike bacteria, fungal communities showed significant compositional differentiation along the restoration sequence, and environmental factors explained the highest proportion of their composition; however, assembly processes remained consistently dominated by drift, a pattern corroborated by the tree-free tNST analysis (**Supplementary Table 3**). This seemingly paradoxical phenomenon illustrates that community compositional change and assembly mechanism shifts are not synchronized: environmental gradients can alter dominant taxa without necessarily changing the drift-dominated assembly background. Fungal communities are substantially influenced by historical contingency, priority effects, and stochastic dispersal processes; the ability of hyphae to span microscale resource patches may also reduce their dependence on local environmental differences (Chase and Myers, 2011; Peay et al., 2016; Jiao et al., 2022). Protistan assembly processes exhibited stronger stage dependence. In AB03, dispersal limitation accounted for up to 90%, which may be related to protist dependence on water film continuity, pore connectivity, and prey resource distribution—physical constraints that had not been sufficiently ameliorated in the early restoration stage (Erktan et al., 2020; Esteban and Fenchel, 2020). As restoration progressed, dispersal limitation weakened while drift strengthened, suggesting that spatial constraint alleviation led to increased roles of random colonization and local extinction. The re-emergence of homogenizing dispersal at AB31 may reflect enhanced community exchange among sites driven by environmental convergence in the later stage (Stegen et al., 2015). Such stage-dependent shifts in assembly mechanisms were not observed in bacteria or fungi, suggesting that protists possess unique sensitivity to changes in environmental connectivity (Luan et al., 2020).

### 4.3 Differential restructuring of microbial co-occurrence networks

Network complexity responses to restoration were asynchronous and group-specific: fungal networks expanded in topological scale in the middle and late restoration stages, bacterial networks showed only a transient dip at AB03, and protistan networks remained relatively stable. The response of fungal networks was mainly manifested as increases in path length and diameter, rather than synchronous enhancement of local connectivity density (average degree) or clustering coefficient. This may be attributable to the ability of hyphae to span soil micro-patches and connect spatially dispersed substrates; consequently, as resource availability improves, their interactions are more likely to expand along heterogeneous resource patches, manifesting as topological scale extension rather than greater local connectivity (Aguilar-Trigueros et al., 2022). However, increases in average path length and diameter do not necessarily equate to functional enhancement, but more likely reflect changes in the spatial organization of networks (Barberán et al., 2012; Banerjee et al., 2018). In contrast, bacterial networks responded more weakly than fungal networks; although homogenizing dispersal was significantly enhanced in the middle restoration stage, network complexity indices did not increase synchronously. Bacteria lack hyphal-mediated active spatial extension capabilities; their dispersal and interactions depend more on water, particle migration, and microscale spatial connectivity (Aguilar-Trigueros et al., 2022). Under enhanced homogenizing dispersal, compositional homogenization among sites may also weaken the spatial differentiation of network structure, preventing effective translation of assembly process changes into enhanced network complexity (Yang et al., 2025).

Protistan networks further demonstrated an asynchronous relationship between assembly processes and network complexity. Although protistan assembly processes changed markedly across restoration stages, the composite network complexity index showed no clear stage-specific differentiation, suggesting that shifts in assembly processes and local taxonomic turnover did not necessarily translate into proportional restructuring of overall network topology. This apparent stability may reflect replacement among taxa occupying similar network positions or compensatory association patterns among protistan groups, which could help maintain overall connectivity during community turnover (Puente-Sánchez et al., 2024; Bodur et al., 2025). Therefore, compared with bacteria and fungi, protistan network topology appeared relatively stable at the whole-network level, even when stage-dependent changes occurred in community assembly.

### 4.4 Microbial group-specific pathways maintaining soil multifunctionality

The divergence between fungal diversity-dependence and bacteria/protistan network-dependence likely reflects fundamental differences in their functional realization modes. Microbial diversity is typically associated with functional complementarity, resource use differentiation, and functional redundancy, whereas network complexity more strongly characterizes the potential association structure and organizational pattern of communities (Langenheder et al., 2010; Puente-Sánchez et al., 2024). In this study, the contribution of diversity to SMF was inconsistent among the three microbial groups, which may be related to their life-history traits, nutritional modes, and interaction structures (Zhang et al., 2026). This is consistent with H1.

The positive relationship between fungal diversity and SMF may reflect fungal functional differentiation in complex organic matter decomposition, extracellular enzyme secretion, and nutrient transformation (Xu et al., 2025). Different fungal groups vary in their ability to utilize substrates such as cellulose, hemicellulose, and lignin; higher diversity broadens the potential substrate utilization range, enabling more decomposition and nutrient transformation processes to be incorporated into the community functional pool (Floudas et al., 2020; Kaschper et al., 2026). This diversity-related pathway is echoed by evidence from the Qinghai–Tibetan Plateau: fungal diversity was repeatedly identified as a key driver of SMF during alpine grassland restoration (Duan et al., 2023; Meng et al., 2026), whereas bacterial diversity was unrelated or even negatively related to SMF (Wang et al., 2021; Meng et al., 2026). Similar findings in both regions suggest that this pathway is a shared feature of alpine grassland restoration. Notably, even under drift dominated assembly, diversity can still reflect the reservoir of potential functional groups in the current community (Qiu et al., 2026), so its functional significance is not diminished where stochastic assembly prevails. However, this negative path captures only the component of network expansion that is independent of diversity: when the variance shared between the two attributes was apportioned between them, both contributed positively to SMF (**Supplementary Table 1**). This residual component—mainly longer paths and larger diameters at the later stages (**Supplementary Table 4**)—may have diluted local metabolic complementarity, as co-occurrence edges largely reflect shared environmental preferences rather than functional linkages (Barberán et al., 2012), accounting for the negative association. These results support H1: fungal associations with SMF were primarily diversity-related.

Although bacterial diversity did not show a significant contribution to SMF, its network complexity had a positive effect. This pattern aligns with the functional realization mode of bacteria: when participating in carbon decomposition, nitrogen transformation, and phosphorus activation, bacteria often require multiple functional groups to sequentially complete substrate decomposition, metabolic intermediate utilization, and nutrient transformation; metabolic complementarity and resource transfer among groups may better reflect the continuity of these processes than a single diversity index (Hu et al., 2025b; Moraïs et al., 2026). The lack of significant effects of bacterial diversity may be related to the homogenizing dispersal dominated assembly pattern, under which community convergence among sites weakens the sensitivity of diversity as a functional indicator. Protists also exhibited positive associations with network complexity, whereas diversity itself did not show positive effects. As important regulators of microbial food webs, protists can alter microbial community structure through predation on bacteria or fungi, indirectly influencing nutrient mineralization processes; such effects may be more reflected in potential trophic linkages and network structures than in species number per se (Zhang et al., 2025). At the trophic-mode level, no diversity metric (richness or Shannon) was significantly correlated with SMF (**Supplementary Table 5**); the negative diversity–SMF path is thus unlikely to reflect conflicting functions among broad trophic modes, but rather functional heterogeneity within these modes. Grazing by predatory protists generally stimulates nutrient mineralization through the microbial loop (Clarholm, 1985); together with the positive path of network complexity, this suggests that the protistan contribution to SMF depends on intact trophic cascades within the network rather than on species number itself.

Collectively, the transmission of assembly changes to SMF depends on the functional realization mode of each group: assembly changes were accompanied by network restructuring, but this cascading effect was strongly group-specific, with fungal diversity, bacterial potential metabolic interactions, and protistan potential trophic linkages representing three distinct pathways linking microorganisms to SMF during abandonment restoration. Such paths may themselves be partly plant-mediated: in an alpine meadow, plant biomass influenced multifunctionality indirectly through arbuscular mycorrhizal fungi (Mao et al., 2024), an indirect pathway that most likely overlaps with the fungal diversity–SMF path. The bacterial and protistan network-complexity paths are less directly tied to vegetation, although plant effects may still reach them through soil properties. The paths identified here are therefore best interpreted as the microbially mediated components of plant–soil–microbe coupling during restoration. Fitted to soil measurements alone, however, our models cannot partition these feedbacks explicitly; the soil-property pathway should thus be read as integrating the cumulative outcome of plant–soil interactions, rather than as a purely abiotic filter. Coupled plant–soil measurements would allow such feedbacks to be separated directly.

## 5 Conclusion

This study analyzed the relationships of bacterial, fungal, and protistan diversity, community assembly, and network complexity with SMF along a restoration sequence from AG to AB31 in an alpine grassland. SMF increased significantly at AB12 and remained elevated through AB31, indicating that restoration gains were concentrated mainly in the early-to-middle stages. Mechanistically, the associations between the three microbial groups and SMF exhibited a strategic differentiation between “diversity-dependence” and “network-dependence”: fungi were characterized mainly by positive diversity associations, whereas bacteria and protists were mainly characterized by positive network complexity associations. This differentiation was coupled with group-specific changes in community assembly processes: enhanced homogenizing dispersal in bacteria during the middle restoration stage, persistent drift dominance in fungi, and a transition from dispersal limitation to drift in protists. These results suggest that alpine grassland restoration assessments should adopt group-specific microbial indicators—fungal Shannon diversity and bacterial and protistan network complexity—instead of universal metrics. Future meta-analyses across different grassland ecosystems could test whether this diversity–network differentiation is generalizable. Metagenomic sequencing along this chronosequence could verify whether these indicators predict the abundances of carbon- and nitrogen-cycling genes.

## Data Availability

The original contributions presented in the study are included in the article/**Supplementary Material**. Further inquiries can be directed to the corresponding authors.
