# Supplementary material for: Divergent group-specific pathways of microbial diversity and network complexity maintain soil multifunctionality during alpine grassland restoration
Source: Front Plant Sci. 2026 Aug 19;17:1923179. doi: 10.3389/fpls.2026.1923179 (PMC13535780; doi:10.3389/fpls.2026.1923179)
Supplement: Supplementary file 1 [file DataSheet1.docx]

Supplementary Material

**Divergent group-specific pathways of microbial diversity and network complexity maintain soil multifunctionality during alpine grassland restoration**

**Fig. S1** Overview map of the study area.

**Fig. S2.** Monthly air temperature (A) and precipitation (B) in the study area: 1984–2024 mean (line) ± 1 SD (shading) versus the 2025 sampling year (red).

**Fig. S3** Drivers of the first three dimensions of multidimensional multifunctionality ((A) Dim1, (B) Dim2, and (C) Dim3) during natural restoration of abandoned alpine grassland. Bar plots show Spearman’s ρ coefficients between individual functions and the principal coordinate analysis (PCoA) axes derived from 14 standardized functions; percentages in parentheses indicate the proportion of variance explained by each PCoA axis.

**Fig. S4** Changes in Shannon diversity index of bacterial, fungal, and protistan communities across restoration stages.

**Fig. S5** Changes in the relative abundance of protistan trophic modes across restoration stages.

**Fig. S6** Redundancy analysis (RDA) revealing the effects of environmental factors on community composition of bacteria (A), fungi (B), and protists (C).

**Fig. S7** Within-group bacterial β-diversity across restoration stages (A; PERMDISP based on Bray–Curtis dissimilarities) and its relationship with soil multifunctionality (B; Spearman correlation).

**Fig. S8** Single-function responses during natural restoration of abandoned alpine grassland. Different lowercase letters above boxplots indicate significant differences among treatments (*P* < 0.05, Tukey’s HSD test).

**Fig. S9** Multidimensional multifunctionality characteristics across restoration stages. (A-C) Responses of the first three dimensions of multidimensional multifunctionality indices (Dim1, Dim2, and Dim3) to restoration treatments. Different lowercase letters above boxplots indicate significant differences among treatments (*P* < 0.05, Tukey’s HSD test).

**Fig. S10** Multi-threshold multifunctionality during natural restoration of abandoned alpine grassland. (A-E) Multi-threshold multifunctionality at five thresholds (10%, 25%, 50%, 75%, and 90% of the maximum value for each function), expressed as the proportion of functions exceeding each threshold. (F) Area under the multi-threshold multifunctionality curve (MUF) across restoration treatments. (*P* < 0.05, Tukey’s HSD test).

**Fig. S11** Sensitivity of soil multifunctionality (SMF) to indicator selection and weighting algorithms. (A) Loadings of the 14 indicators on the first principal component. (B) Stage patterns under three SMF variants: original SMF, SMF excluding total potassium, and the first principal component of the multidimensional metric. (C) Relationships between fungal network complexity and SMF under the same variants.

**Table S1.** Diagnostics of the PLS-PM models: variance inflation factors (VIF) of all inner-model equations, residual diagnostics of the SMF equations, and relative-weights (LMG) decomposition of the fungal SMF equation (R² = 0.42).

**Table S2.** One-way ANOVA of soil microbial α-diversity indices and protistan trophic-mode attributes across restoration stages. Values are mean ± SE (n = 5); significant effects (*P* < 0.05) are shown in bold.

**Table S3** Taxonomic normalized stochasticity ratio (tNST) test for fungal communities across restoration stages.

**Table S4** One-way ANOVA results (F, df, P) for microbial network topological indices across restoration stages. Values are means ± SE (n = 5 per stage). Different letters indicate significant differences (Tukey's HSD, *P* < 0.05).

**Table S5** Spearman correlation coefficients between protist trophic mode attributes and soil multifunctionality indices (MFI, MDF, MUF). Values are correlation coefficients (ρ) with *P*‑values in parentheses.

**Table S6** Sensitivity of soil multifunctionality (SMF) to indicator selection and weighting algorithms.

**Table S7** Correlation analysis between different soil multifunctionality (SMF) variants and potential driving factors.

**Fig. S1** Overview map of the study area.

**Fig. S2.** Monthly air temperature (A) and precipitation (B) in the study area: 1984–2024 mean (line) ± 1 SD (shading) versus the 2025 sampling year (red).

**Fig. S3** Drivers of the first three dimensions of multidimensional multifunctionality ((A) Dim1, (B) Dim2, and (C) Dim3) during natural restoration of abandoned alpine grassland. Bar plots show Spearman’s ρ coefficients between individual functions and the principal coordinate analysis (PCoA) axes derived from 14 standardized functions; percentages in parentheses indicate the proportion of variance explained by each PCoA axis.

**Fig. S4** Changes in Shannon diversity index of bacterial, fungal, and protistan communities across restoration stages.

**Fig. S5** Changes in the relative abundance of protistan trophic modes across restoration stages.

**Fig. S6** Redundancy analysis (RDA) revealing the effects of environmental factors on community composition of bacteria (A), fungi (B), and protists (C).

**Fig. S7** Within-group bacterial β-diversity across restoration stages (A; PERMDISP based on Bray–Curtis dissimilarities) and its relationship with soil multifunctionality (B; Spearman correlation).

**Fig. S8** Single-function responses during natural restoration of abandoned alpine grassland. Different lowercase letters above boxplots indicate significant differences among treatments (*P* < 0.05, Tukey’s HSD test).

**Fig. S9** Multidimensional multifunctionality characteristics across restoration stages. (A-C) Responses of the first three dimensions of multidimensional multifunctionality indices (Dim1, Dim2, and Dim3) to restoration treatments. Different lowercase letters above boxplots indicate significant differences among treatments (*P* < 0.05, Tukey’s HSD test).

**Fig. S10** Multi-threshold multifunctionality during natural restoration of abandoned alpine grassland. (A-E) Multi-threshold multifunctionality at five thresholds (10%, 25%, 50%, 75%, and 90% of the maximum value for each function), expressed as the proportion of functions exceeding each threshold. (F) Area under the multi-threshold multifunctionality curve (MUF) across restoration treatments. (*P* < 0.05, Tukey’s HSD test).

**Fig. S11** Sensitivity of soil multifunctionality (SMF) to indicator selection and weighting algorithms. (A) Loadings of the 14 indicators on the first principal component. (B) Stage patterns under three SMF variants: original SMF, SMF excluding total potassium, and the first principal component of the multidimensional metric. (C) Relationships between fungal network complexity and SMF under the same variants.

| **Table S1. Diagnostics of the PLS-PM models: variance inflation factors (VIF) of all inner-model equations, residual diagnostics of the SMF equations, and relative-weights (LMG) decomposition of the fungal SMF equation (R² = 0.42).** | | | | | | | | | |
| --- | --- | --- | --- | --- | --- | --- | --- | --- | --- |
| **Model** | **Equation**  **(endogenous construct)** | **Predictor** | **VIF** | **R²** | **Shapiro-Wilk P** | **Heteroscedasticity P** | **Durbin–Watson** | **LMG (averaged R² increment)** | **Share of explained variance (%)** |
| **Bacterial** | Soil physicochemical | Succession time | 1 |  |  |  |  |  |  |
|  | Bacterial βNTI | Succession time | 3.45 |  |  |  |  |  |  |
|  | Bacterial βNTI | Soil physicochemical | 3.45 |  |  |  |  |  |  |
|  | Bacterial diversity | Soil physicochemical | 1 |  |  |  |  |  |  |
|  | Bacterial diversity | Bacterial βNTI | 1 |  |  |  |  |  |  |
|  | Network complexity | Succession time | 3.77 |  |  |  |  |  |  |
|  | Network complexity | Soil physicochemical | 3.62 |  |  |  |  |  |  |
|  | Network complexity | Bacterial βNTI | 1.07 |  |  |  |  |  |  |
|  | Network complexity | Bacterial diversity | 1.29 |  |  |  |  |  |  |
|  | SMF | Succession time | 3.53 |  |  |  |  |  |  |
|  | SMF | Soil physicochemical | 3.63 |  |  |  |  |  |  |
|  | SMF | Bacterial diversity | 1.32 |  |  |  |  |  |  |
|  | SMF | Network complexity | 1.2 |  |  |  |  |  |  |
|  | **SMF equation (residual diagnostics)** | |  | **0.455** | **0.936** | **0.582** | **1.51** |  |  |
| **Fungal** | Soil physicochemical | Succession time | 1 |  |  |  |  |  |  |
|  | Fungal βNTI | Succession time | 4.5 |  |  |  |  |  |  |
|  | Fungal βNTI | Soil physicochemical | 4.5 |  |  |  |  |  |  |
|  | Fungal diversity | Soil physicochemical | 1 |  |  |  |  |  |  |
|  | Fungal diversity | Fungal βNTI | 1 |  |  |  |  |  |  |
|  | Network complexity | Succession time | 4.5 |  |  |  |  |  |  |
|  | Network complexity | Soil physicochemical | 5.09 |  |  |  |  |  |  |
|  | Network complexity | Fungal βNTI | 1.39 |  |  |  |  |  |  |
|  | Network complexity | Fungal diversity | 1.93 |  |  |  |  |  |  |
|  | SMF | Succession time | 5.92 |  |  |  |  | 0.1484 | 35 |
|  | SMF | Soil physicochemical | 8.16 |  |  |  |  | 0.1775 | 41.9 |
|  | SMF | Fungal diversity | 9.22 |  |  |  |  | 0.0647 | 15.3 |
|  | SMF | Network complexity | 7.48 |  |  |  |  | 0.0331 | 7.8 |
|  | **SMF equation (residual diagnostics)** | |  | **0.424** | **0.547** | **0.758** | **1.20** |  |  |
| **Protistan** | Soil physicochemical | Succession time | 1.01 |  |  |  |  |  |  |
|  | Protistan βNTI | Succession time | 1.81 |  |  |  |  |  |  |
|  | Protistan βNTI | Soil physicochemical | 1.81 |  |  |  |  |  |  |
|  | Protistan diversity | Soil physicochemical | 1.11 |  |  |  |  |  |  |
|  | Protistan diversity | Protistan βNTI | 1.11 |  |  |  |  |  |  |
|  | Network complexity | Succession time | 2.96 |  |  |  |  |  |  |
|  | Network complexity | Soil physicochemical | 2.99 |  |  |  |  |  |  |
|  | Network complexity | Protistan βNTI | 1.17 |  |  |  |  |  |  |
|  | Network complexity | Protistan diversity | 1.76 |  |  |  |  |  |  |
|  | SMF | Succession time | 2.96 |  |  |  |  |  |  |
|  | SMF | Soil physicochemical | 3.17 |  |  |  |  |  |  |
|  | SMF | Protistan diversity | 2.05 |  |  |  |  |  |  |
|  | SMF | Network complexity | 1.41 |  |  |  |  |  |  |
|  | **SMF equation (residual diagnostics)** | |  | **0.434** | **0.773** | **0.705** | **1.43** |  |  |

Note: Residual diagnostics (R², Shapiro–Wilk *P*, heteroscedasticity *P*, and Durbin–Watson statistic) were computed for the SMF equation of each model. LMG relative-weights decomposition was performed for the fungal SMF equation only, in which diversity and network complexity were strongly correlated; blank cells indicate not applicable.

| **Table S2. One-way ANOVA of soil microbial α-diversity indices and protistan trophic-mode attributes across restoration stages.** Values are mean ± SE (n = 5); significant effects (*P* < 0.05) are shown in bold. | | | | | | | | |
| --- | --- | --- | --- | --- | --- | --- | --- | --- |
|  | **Metric** | **AG** | **AB03** | **AB12** | **AB31** | **F** | **df** | **P** |
| Bacteria | Richness | 2173.8 ± 44.32a | 2014 ± 60.92a | 1946.6 ± 16.48a | 1950.4 ± 101.65a | 2.78 | 3, 16 | **0.075** |
|  | Shannon | 6.88 ± 0.04a | 6.83 ± 0.07a | 6.78 ± 0.01a | 6.73 ± 0.06a | 1.59 | 3, 16 | 0.2303 |
| Fungi | Richness | 394.4 ± 64.01a | 435.2 ± 20.29a | 493.4 ± 36.81a | 532.2 ± 45.77a | 1.87 | 3, 16 | 0.1746 |
|  | Shannon | 3.96 ± 0.2a | 4.3 ± 0.06a | 4.41 ± 0.06a | 4.17 ± 0.06a | 2.76 | 3, 16 | **0.0762** |
| Protist | Richness | 32.4 ± 3.04a | 28.2 ± 1.93a | 35.2 ± 4.54a | 28 ± 2.7a | 1.19 | 3, 16 | 0.3462 |
|  | Shannon | 2.75 ± 0.06a | 2.33 ± 0.07b | 2.63 ± 0.07ab | 2.37 ± 0.14b | 5.06 | 3, 16 | **0.0118** |
| Predatory | Richness | 10.400 ± 1.249 | 12.800 ± 0.860 | 14.600 ± 2.159 | 8.000 ± 1.000 | 4.15 | 3, 16 | **0.0237** |
|  | Shannon | 1.701 ± 0.074 | 1.740 ± 0.108 | 1.973 ± 0.084 | 1.245 ± 0.141 | 8.43 | 3, 16 | **0.0014** |
|  | Relative abundance | 0.456 ± 0.058 | 0.772 ± 0.024 | 0.715 ± 0.018 | 0.533 ± 0.070 | 9.62 | 3, 16 | **0.0007** |
| Phototrophic | Richness | 6.000 ± 0.837 | 3.800 ± 0.490 | 5.400 ± 0.678 | 4.400 ± 0.400 | 2.5 | 3, 16 | 0.0969 |
|  | Shannon | 1.354 ± 0.160 | 1.108 ± 0.089 | 1.382 ± 0.142 | 1.144 ± 0.033 | 1.45 | 3, 16 | 0.2644 |
|  | Relative abundance | 0.128 ± 0.026 | 0.032 ± 0.007 | 0.057 ± 0.002 | 0.102 ± 0.051 | 2.3 | 3, 16 | 0.1159 |
| Parasitic | Richness | 16.000 ± 2.387 | 11.600 ± 1.166 | 15.200 ± 2.035 | 15.600 ± 2.112 | 1.05 | 3, 16 | 0.398 |
|  | Shannon | 1.981 ± 0.179 | 1.613 ± 0.088 | 1.781 ± 0.148 | 1.939 ± 0.191 | 1.14 | 3, 16 | 0.3624 |
|  | Relative abundance | 0.417 ± 0.076 | 0.196 ± 0.027 | 0.228 ± 0.018 | 0.365 ± 0.048 | 4.92 | 3, 16 | **0.0131** |

| **Table S3 Taxonomic normalized stochasticity ratio (tNST) test for fungal communities across restoration stages.** | | | | |
| --- | --- | --- | --- | --- |
| **Stage** | **Observed Bray–Curtis (D_obs)** | **Null Bray–Curtis (mean ± SD)** | **tNST (%)** | **Interpretation** |
| AG | 0.548 | 0.322 ± 0.065 | 66.7 | stochasticity dominates (>50%) |
| AB03 | 0.463 | 0.272 ± 0.012 | 63.1 | stochasticity dominates (>50%) |
| AB12 | 0.487 | 0.278 ± 0.010 | 64.5 | stochasticity dominates (>50%) |
| AB31 | 0.513 | 0.195 ± 0.011 | 69.8 | stochasticity dominates (>50%) |

Note: tNST was calculated based on abundance‑weighted Bray-Curtis dissimilarities using the proportional‑fixed (PF) null model with 1,000 randomizations. tNST > 50% indicates that stochastic processes dominate community assembly.

| **Table S4 One-way ANOVA results (F, df, P) for microbial network topological indices across restoration stages. Values are means ± SE (n = 5 per stage). Different letters indicate significant differences (Tukey's HSD, *P* < 0.05).** | | | | | | | | |
| --- | --- | --- | --- | --- | --- | --- | --- | --- |
| **Microbe** | **Metric** | **AG** | **AB03** | **AB12** | **AB31** | **F** | **df** | **P** |
| Bacteria | Nodes | 394.2 ± 9.3776a | 419.8 ± 3.8a | 406.6 ± 4.4452a | 392.6 ± 16.7111a | 1.596 | 3, 16 | 0.2296 |
|  | Edges | 2348 ± 69.4125a | 2581.2 ± 45.9199a | 2479.2 ± 39.1719a | 2263 ± 170.7527a | 2.102 | 3, 16 | 0.1402 |
|  | Average degree | 11.9077 ± 0.1195a | 12.294 ± 0.1198a | 12.1931 ± 0.0944a | 11.4576 ± 0.4403a | 2.426 | 3, 16 | 0.1034 |
|  | Clustering coefficient | 0.4532 ± 0.008a | 0.4547 ± 0.0062a | 0.4675 ± 0.0037a | 0.4617 ± 0.0028a | 1.415 | 3, 16 | 0.2751 |
|  | Average path length | 3.6316 ± 0.0281a | 3.6385 ± 0.0198a | 3.622 ± 0.0168a | 3.6887 ± 0.0149a | 2.103 | 3, 16 | 0.1401 |
|  | Diameter | 10 ± 0.3162a | 10.2 ± 0.2a | 9.6 ± 0.2449a | 10.2 ± 0.2a | 1.333 | 3, 16 | 0.2985 |
|  | Density | 0.0303 ± 7e-04a | 0.0294 ± 2e-04a | 0.0301 ± 3e-04a | 0.0293 ± 4e-04a | 1.398 | 3, 16 | 0.2798 |
| Fungi | Nodes | 121.6 ± 9.626a | 137 ± 2.2361a | 133.2 ± 3.4264a | 141.6 ± 4.5782a | 2.245 | 3, 16 | 0.1225 |
|  | Edges | 154 ± 19.1833a | 189 ± 15.7321a | 175.2 ± 17.3793a | 196 ± 16.0686a | 1.165 | 3, 16 | 0.3537 |
|  | Average degree | 2.4949 ± 0.1222a | 2.761 ± 0.2295a | 2.6106 ± 0.1972a | 2.7499 ± 0.1559a | 0.488 | 3, 16 | 0.6956 |
|  | Clustering coefficient | 0.4229 ± 0.0233ab | 0.4548 ± 0.0188a | 0.4096 ± 0.0168ab | 0.3781 ± 0.0129b | 3.006 | 3, 16 | 0.0612 |
|  | Average path length | 3.9162 ± 0.1532c | 4.4069 ± 0.133bc | 5.2079 ± 0.1345a | 4.9003 ± 0.2826ab | 9.24 | 3, 16 | **<0.001** |
|  | Diameter | 10 ± 0.4472c | 10.4 ± 0.5099bc | 14.2 ± 0.6633a | 12.6 ± 0.6ab | 12.275 | 3, 16 | **<0.001** |
|  | Density | 0.0209 ± 7e-04a | 0.0203 ± 0.0018a | 0.0196 ± 0.001a | 0.0195 ± 6e-04a | 0.332 | 3, 16 | 0.802 |
| Protist | Nodes | 22.2 ± 0.7348a | 19.4 ± 1.0296a | 23.6 ± 2.358a | 20.6 ± 1.8601a | 1.269 | 3, 16 | 0.3184 |
|  | Edges | 37 ± 10.3586a | 45.6 ± 7.5934a | 48 ± 10.015a | 21.4 ± 4.3428a | 2.038 | 3, 16 | 0.1491 |
|  | Average degree | 3.4019 ± 1.0214a | 4.6882 ± 0.7383a | 3.956 ± 0.5922a | 2.0253 ± 0.3203a | 2.483 | 3, 16 | 0.098 |
|  | Clustering coefficient | 0.9938 ± 0.0062a | 0.9747 ± 0.0161a | 0.9913 ± 0.0053a | 0.9502 ± 0.0231a | 1.869 | 3, 16 | 0.1755 |
|  | Average path length | 1.0389 ± 0.0389a | 1.0509 ± 0.0356a | 1.0755 ± 0.0509a | 1.2409 ± 0.1678a | 1.057 | 3, 16 | 0.3948 |
|  | Diameter | 1.2 ± 0.2a | 1.6 ± 0.4a | 1.4 ± 0.2449a | 2.2 ± 0.4899a | 1.493 | 3, 16 | 0.2543 |
|  | Density | 0.1646 ± 0.0529ab | 0.2573 ± 0.0435a | 0.1781 ± 0.0213ab | 0.1039 ± 0.0126b | 3.000 | 3, 16 | 0.0615 |

| **Table S5 Spearman correlation coefficients between protist trophic mode attributes and soil multifunctionality indices (MFI, MDF, MUF). Values are correlation coefficients (ρ) with *P*‑values in parentheses.** | | | | |
| --- | --- | --- | --- | --- |
| **Trophic mode** | **Metric** | **MFI** | **MDF** | **MUF** |
| Predatory | Richness | -0.066 (P = 0.783) | -0.177 (P = 0.456) | -0.032 (P = 0.894) |
|  | Shannon | 0.018 (P = 0.940) | 0.161 (P = 0.498) | 0.009 (P = 0.970) |
|  | Relative abundance | 0.313 (P = 0.179) | -0.301 (P = 0.198) | 0.358 (P = 0.121) |
| Phototrophic | Richness | -0.213 (P = 0.366) | 0.289 (P = 0.217) | -0.220 (P = 0.352) |
|  | Shannon | -0.020 (P = 0.935) | 0.286 (P = 0.222) | 0.017 (P = 0.945) |
|  | Relative abundance | -0.214 (P = 0.366) | 0.564 (P = 0.010) | -0.254 (P = 0.280) |
| Parasitic | Richness | -0.098 (P = 0.682) | 0.209 (P = 0.377) | -0.144 (P = 0.546) |
|  | Shannon | -0.059 (P = 0.806) | 0.084 (P = 0.724) | -0.092 (P = 0.701) |
|  | Relative abundance | -0.173 (P = 0.466) | 0.200 (P = 0.398) | -0.217 (P = 0.359) |

| **Table S6 Sensitivity of soil multifunctionality (SMF) to indicator selection and weighting algorithms.** | | | | | | | |
| --- | --- | --- | --- | --- | --- | --- | --- |
| **SMF version** | **ANOVA P** | **Bacterial  network complexity** | **Fungal  network complexity** | **Protistan  network complexity** | **Bacterial  Shannon** | **Fungal  Shannon** | **Protistan  Shannon** |
| SMF (original) | 0.0001 | 0.09 | +0.51* | -0.01 | -0.19 | 0.35 | -0.34 |
| Excluding TK | 0 | 0.13 | +0.57* | -0.15 | -0.32 | 0.31 | -0.29 |
| PC1 | 0 | 0.25 | 0.36 | -0.04 | -0.06 | 0.25 | 0.43 |

Note: Three SMF variants were compared: the original SMF (with 14 indicators), SMF without the TK indicator (with 13 indicators), and the first principal component (PC1). The table presents one‑way ANOVA results for each variant across the four restoration stages, as well as Spearman correlation coefficients between each variant and network complexity, and Shannon diversity of three microbial groups.

| **Table S7 Correlation analysis between different soil multifunctionality (SMF) variants and potential driving factors.** | | | | | | | | |
| --- | --- | --- | --- | --- | --- | --- | --- | --- |
| **SMF version** | **Succession**  **time** | **Soil properties** | **Bacterial**  **βNTI** | **Fungal**  **βNTI** | **Protistan**  **βNTI** | **Bacterial**  **richness** | **Fungal**  **richness** | **Protistan**  **richness** |
| SMF (original) | +0.76* | +0.55* | -0.43 | -0.02 | -0.36 | -0.23 | 0.26 | -0.11 |
| MUF | +0.73* | +0.50* | -0.48* | -0.06 | -0.39 | -0.23 | 0.2 | -0.13 |
| SMF excluding TK | +0.84* | +0.71* | -0.34 | 0.11 | -0.19 | -0.31 | 0.33 | -0.15 |
| PC1 (MDF) | 0.28 | +0.81* | 0.13 | -0.06 | 0.14 | -0.07 | 0.23 | 0.1 |

Note: Values are Spearman correlation coefficients (ρ); * indicates *P* < 0.05. The SMF variants include the original SMF (14 indicators), MUF (multifunctionality index), SMF excluding TK (13 indicators), and PC1 (PCA axis 1). Driving factors encompass restoration time, soil properties (PC1), βNTI (phylogenetic turnover index) of three microbial groups, and their richness.
